# The Pathogenesis and Treatment of Emotion Dysregulation in Borderline Personality Disorder

**DOI:** 10.1155/2015/179276

**Published:** 2015-08-26

**Authors:** Andreas Laddis

**Affiliations:** ^1^Private Practice, Framingham, MA 01545, USA; ^2^School of Public Health, Boston University, Boston, MA 02118, USA; ^3^International Society for the Study of Trauma and Dissociation, McLean, VA 22102, USA

## Abstract

Uncontrollable emotional lability and impulsivity are a paramount phenomenon of Borderline Personality Disorder (BPD). This paper aims to review theories that entertain emotion dysregulation as the core deficit of BPD and a key factor in the etiology of BPD, in order, then, to propose the author's own theory, which arguably transcends certain limitations of the earlier ones. The author asserts that his psychodynamic theory explains the symptoms of BPD more thoroughly and it inspires a more parsimonious interpretation of brain imaging findings. In closing, the author draws implications of the proposed theory for clinical practice. He reports an efficacy study for treatment of emotion dysregulation based on that theory.

## 1. Introduction

The generic term “emotion dysregulation” is used often to characterize a range of behavioral phenomena that are paramount in Borderline Personality Disorder (BPD). These phenomena consist of various negative emotions, a succession of fear, anger, anxiety, depression, guilt, and shame, with uncontrollable intensity and duration. Sufferers report feeble will to contain these emotions or to engage with others' interventions to that end [1]. Dysregulation of negative emotions occurs in the larger context of mood lability, that is, shifting abruptly between negative and positive moods, although negative moods still dominate [1, 2].

The term “emotion dysregulation” is also used for etiological constructs, explanations for this uncontrollability of emotions. Mostly, etiological theories are biological. They postulate an innate limbic abnormality and, with less certainty, a corollary abnormality in the prefrontal cortex [3–7]. They are inspired by the unresponsiveness of emotion dysregulation in BPD to psychotherapeutic interventions and by the resemblance to emotion dysregulation in certain neurological syndromes. The most accepted variant among them [3] proposes that limbic abnormality is not sufficient to explain the pathogenesis of emotion dysregulation. It adds the qualification that, differently from other neurological abnormalities, it is possible to learn skills to modulate the BPD kind of abnormal limbic excitation, just as it is for normal excitation. A severe limitation of biological theories is failure to explain how a limbic abnormality can be object-specific. In contrast to neurological syndromes, emotion dysregulation in BPD occurs only with negative emotions evoked strictly during adversity in a relationship, like threat of betrayal or abandonment [2, 8]. Even so, it does not occur in every instance of such adversity [1, 7]. More ambitiously, psychodynamic theories about the nature of emotion dysregulation in BPD aim to transcend that limitation of biological theories.

This paper begins with review of factor analyses of BPD symptoms, which strongly indicate that emotion dysregulation is a cardinal feature of BPD. Next, it reviews studies in the neuropsychology and neurobiology of normal emotion regulation and its developmental roots in attachment theory. The next two sections review biological and psychodynamic theories of emotion dysregulation in BPD. That review concludes with presentation of the author's own psychodynamic theory that is composed of well-researched concepts from cognitive psychology. The proposed theory features a very specific core psychological shortcoming born of errant, sometimes exploitative, caretaking practices in BPD patients' childhood. The paper proceeds with description of clinical concepts and techniques that implement the proposed theory's advantages and with a preliminary study of such interventions' efficacy.

## 2. Descriptive Psychopathology and Factor Analyses

Exploratory and confirmatory factor analyses of diagnostic criteria for BPD support the hypothesis that emotion dysregulation is the core mechanism [9–14]. These factor analyses used diagnostic criteria from the American Psychiatric Association (APA)* Diagnostic and Statistical Manual of Mental Disorders*,* 4th Edition* (DSM-IV) [2]. (The criteria remain unchanged in the current edition, DSM-5.) The analyses conclude in favor of either a one-factor or a three-factor model. Emotion dysregulation pertains to eight of nine diagnostic criteria (exception: chronic feelings of emptiness) [2, p. 654]. They are rephrased below to highlight the strict correlation of emotional excess and lability with very specific adversity, namely, threat of betrayal or abandonment:

- Frantic efforts to avoid real or imagined abandonment in a valuable relationship.
- Lability, that is, sudden and dramatic shifts in the sufferer's view of oneself and of others, alternating between idealization and devaluation.
- Affective instability, marked reactivity of mood, mainly irritability and anxiety.
- Potentially self-damaging impulsivity, for example, reckless sex or driving, drug use.
- Inappropriate and intense, uncontrollable anger.
- Recurrent threats to commit suicide or self-mutilation.
- Transient, stress-related paranoia and dissociative experiences.

Several studies revealed a unidimensional structure for BPD [10, 11, 14]. Among them, Fossati et al. [10] as well as Johansen et al. [14] found high diagnostic efficiency of “unstable relationships,” which highlights the object-specificity of emotion dysregulation in BPD. Others [9, 12, 13] found support for multidimensional models. One exploratory factor analysis [9] revealed three factors, disturbed identity and interpersonal relationships, affective dysregulation, and impulsivity. Sanislow and colleagues [12] submitted this model to confirmatory factor analysis. They found DSM-IV criteria to comprise a statistically coherent construct, along three dimensions, now named disturbed relatedness, affective dysregulation, and behavioral dysregulation. Putnam and Silk [7] assert that the results from Sanislow et al. can also be interpreted as representing a single factor because those three factors pertain to a single event. Factor analyses with adolescent cohorts [11] showed a small, disputable departure from the construct of BPD for adults.

Findings from studies of the neurobiology of BPD are pertinent to those from factor analyses. Hypotheses for brain imaging studies in BPD are based on findings for normal emotion regulation. As expected, they focus on the prefrontal cortex (PFC), anterior cingulate cortex (ACC), hippocampus, and amygdala and their connections [7, 15]. A thoughtful review concludes that “structural, resting functional, and task-related functional neuroimaging in BPD implicate a network consisting of disrupted amygdala and PFC function, in particular regions in the ACC … and ventral medial PFC” [7, p. 915]. Occasionally, findings for the PFC were negative [16, 17]. Some authors considered their findings to be characteristic of BPD, compared with brain imaging for other disorders with emotion dysregulation. Tebartz van Elst et al. [18] thought so of the specific combination of reduced volume in the amygdala, hippocampus, right ACC, and left orbitofrontal cortex. Others made a similar claim more precisely for reduced activity in the subgenual anterior cingulate cortex [19]. Alternative interpretations for such neurobiological findings in BPD will be discussed in a later section.

## 3. Normal Emotion Regulation and Its Development

### 3.1. Neuropsychology and Cognitive Psychology

The study of emotion regulation is one facet of studying the relation between cognition and emotion. It has attained well-researched concepts about the function and nature of emotion regulation [20–24].

The function of emotion regulation is to “stop and think” [25]. It is to stop pursuit of one's current goal or to suspend one's mood, that is, one's lingering emotional preference for the current goal, as the means to resetting one's priorities. The initiative to reset priorities is carried out more or less deliberately, as a priority of its own value, driven by preference for it. Such preference for resetting priorities is born of (a) reasoning about problems with the current goal (unforeseen costliness, neglect of emergent, competing needs, etc.) and (b) reasoning about alternative priorities becoming valuable and feasible [24, 26]. The* power* to reason about alternative priorities takes leaps with stages of brain maturation. The* will* to rethink one's priorities, on the other hand, is acquired with individual experience of its benefit, that is, the benefit of searching for new possibilities before one can know that they will be found. Infants are introduced to that benefit by caretakers who help them regulate their emotions and guide their attention about possibilities in reality that the children themselves cannot discern.  Section 3.3 will describe progress in the study of attachment and development of emotion regulation in some detail.

Normally, emotions mostly end spontaneously, without the person's awareness of priority when an initiative of changing priorities or when an initiative of different emotional value and higher priority is born in response to developments in reality or in the mind. That entails a physiological representation of the hierarchy of these priorities from old learning. Implicit preferences derive from success with old, once-conscious choices [23, 24]. For similar reasons, the preference to rethink an ongoing commitment itself may take over spontaneously. But shifting emotions spontaneously should not be called “emotion regulation.” The term “emotion regulation” should be reserved for intentional manipulation of (a) triggers of unwanted emotions and of (b) the course of the triggered emotion. Intention to stop and think, in turn, requires finding reasons to do so under the current conditions or adopting a trusted other's reasons.

Similar to the implicit mechanism that resets priorities spontaneously, there is an implicit mechanism that resists changing priorities; it grants force and tenacity to the emotion that drives the current priority [21–23]. This mechanism, too, derives from a once-conscious determination for the importance and urgency of the current goal. Commitment to a goal automatically primes the person's attention selectively for developments, in reality or in the mind, that are pertinent to the completion of this goal, developments conducive or adverse to it. Furthermore, that preference becomes stronger as the person approaches timely completion. Intentional emotion regulation may be initiated on top of these spontaneous adjustments of priority and force, whether to enhance them or counter them.

It is important to cite here yet another implicit psychological influence because it pertains to the review of emotion dysregulation in BPD later. Sometimes emotion seems to be generated and to endure independently from driving a goal, purposelessly. It seems that it starts with no reason, no intended outcome in relation to desired or feared objects, whether in reality or in the mind. That is why clinicians and factor analyses often distinguish between behavior dysregulation and emotion dysregulation. Sometimes, patients feel unable to stop pursuit of a failing goal in order to think of adjustments to its course or to think of priorities to replace it altogether. At other times, patients feel unable to end a seemingly purposeless emotion. They want to modulate it because it colors their current preferences, counter to their conscious reasoning, and it persists irrationally. There are two kinds of such lingering, seemingly purposeless moods. One is a mood of worry and irritability that coincides with rumination about lingering concerns, whose unintended contemplation intrudes recurrently. The second kind is a mood of vigilant anticipation of mistreatment in every relationship. Research in cognitive psychology has shown that such persistent and pervasive moods are initiated by conscious determination of priority to ascertain a current threat of betrayal. Then, they linger and prime attention selectively for negative aspects of unrelated priorities [21].

### 3.2. Neurobiology

The prefrontal cortex (PFC) is unanimously considered the locus of self-organization and self-direction [7, 26, 27]. Miller and Cohen describe the PFC function in concise and insightful terms: It is to “orchestrate thought and action in accordance with internal goals” [26, p. 167]. It consists of “active maintenance of patterns of activity that represent goals and the means to achieve them. They provide bias signals throughout much of the rest of the brain, affecting … sensory modalities, as well as systems responsible for response execution, memory retrieval, emotional evaluation, etc.” [26, p. 171]. The authors properly avoid saying that “it,” the PFC, modulates, inhibits, primes, and so forth the function of other brain organs, because the patterns that the PFC relays to those organs represent the operations and commitments to one or another goal by the activity of the brain as a whole. A special property of the PFC perhaps pertains to the fact that goals gain immunity from interference as they approach completion. The PFC neurons retain the representation of the intent to complete the goal despite processing intervening inputs for other possibilities of initiative [26]. Furthermore, these authors hypothesize, progression to completion strengthens the interaction between neural representations of the effective behavior and neural representations of the transformations in the object. With reiteration of the process for similar goals, memory of successful behavior is encoded as a single skill with subtle and substitutable variations [26]. Miller and Cohen cite evidence that success-related emotions induce connectivity and structural development with neuromodulatory signals from the tegmentum to the PFC.

The insight that the experience of success may correlate with the size of brain organs that organize and encode learning will be revisited while entertaining interpretations of structural brain imaging findings in BPD. It has inspired a similar well-founded hypothesis about the size of the ACC in particular [28]. As mentioned earlier, some consider hypofunction of ACC's subgenual part or small size of the right ACC to be corollary of emotion dysregulation in BPD. The function of the ACC is commonly characterized as “conflict monitoring” because the ACC becomes active during experimental tasks that require a very exact order of steps despite constant cues to follow a more familiar but erroneous order [28]. That creates the subjective experience of vigilance and effort in order to inhibit responding to the familiar cues. How the ACC contributes to success remains in dispute. Certainly, the task requires some form of effortful, repetitive reappraisal and regulation of the familiar preference. Remarkably, this experimental task resembles having to ascertain threat of betrayal, disregarding someone's seductive cues of benevolence that obscure cues of betrayal.

### 3.3. Attachment and Development of Emotion Regulation

The essential social-psychological task of raising children is to develop their ability to reason about their priorities and to know when such reasoning itself becomes a priority. Biologically, that means (a) maturation of working memory, that is, the brain's capacity to manipulate and compare a range of options, including untested possibilities, and (b) the ability to inhibit urges deriving from old preferences. Socially, it means learning to collaborate with others (a) for possibilities of individual initiatives beyond one's own imagination and (b) for ends that can be pursued well only jointly. This section will present briefly the foundation of such development in childhood with an emphasis on emotion regulation.

The physiology of fundamental emotions itself is not fully mature at birth [29]. The complement of characteristics (facial expression, body posture, hormonal, autonomic, etc.) of fundamental emotions is innately given and invariable. Some of them, for example, distress from physical discomfort and disgust at certain smells and tastes, are mature even in preterm babies. Unmistakable expression of anger, on the other hand, seems to coincide with the advent of locomotion. Critical among fundamental emotions are surprise and interest for novelty. Infants' distress from fear manifests differently from distress due to frustration of interest and play [27].

The physiology of comparing a range of options and inhibiting old preferences (frontal and cingulate cortex, hippocampus) matures very slowly, into late adolescence or beyond [30]. Its maturation seems to progress in pace with the growth of the parietal and temporal cortex and certain subcortical and cerebellar regions [29, 31]. That physiological fact is manifested in behavioral progress. Six-month-old infants are already able to inhibit distraction when invited to learn something new [29]. But the capacity for speed, accuracy, and complexity of those operations grows later by means of utilizing information from those concurrently growing regions. The size and functionality of the ACC, especially on the right side, seem to take the largest leap in toddlers [27, 28].

Infants' mature fundamental emotions are the medium for induction of growth by caretakers. Infants spontaneously engage in novelty, but their interest propels them to exploration only to a point. Their motivation must be sustained by caretakers who structure and demonstrate the possibilities for the next step [32], a process aptly called motivational scaffolding [33]. That begins with attunement with the caretaker's facial expression of interest, desire, fear, or whatever, in reference to objects and events. Eventually, children learn to initiate this kind of “social referencing,” looking at the caretaker's face and posture as a guide about what matters around them [29]. Infants become able to disregard interfering temptation, fear, or discomfort, more or less spontaneously, while the caretaker's eyes and gestures direct their interest to an object [33]. If necessary, caretakers may manipulate distracting objects and the infant's attention and posture to create opportune conditions for the child's commitment to the goal. Staging lessons collaboratively in that manner is for children the source of learning the value and the skills for willful, effortful emotion self-regulation.

From the regularity of parents' response to their distress, infants learn to expect being picked up for comfort. One-month-old infants soothe themselves while the caretaker is still approaching [34]. At four months, infants cry more loudly if the caretaker is unresponsive. Beginning at that age, the distress probably is not from physical discomfort but from wanting company for play and exploration [29, 32]. Children, then, may endure frustration in play because “affection/faith in the caretaker is a more important motive than [solitary] pursuit of the goal” [29, p. 519]. Perhaps surprisingly, it seems that before age of six months infants will take comfort from any responder, whereas later they learn to expect it from a preferred caretaker [35].

## 4. Biological Theories and the Neurobiology of BPD

There are two variants of biological explanations of emotion dysregulation in BPD:

1. The first variant is about biological abnormality of limbic excitation, alone or combined with abnormality of the prefrontal regulating cortex. The prototypical, comprehensive developmental theory by Linehan tends to attribute such abnormality to genetics. Others, mainly from the domain of Complex Posttraumatic Stress Disorder (Complex PTSD), an entity related to BPD, with very similar pattern of emotion dysregulation, attribute such abnormality to toxic influences from the physiology of recurrent traumatic distress in childhood [36].
2. The second variant also comes from the domain of Complex PTSD [37]. It postulates that the physiological representation of failed, trauma-related coping is endowed with extraordinary force. When triggered later by reminders of such trauma, the person reflexively resumes the old commitment to successful coping with force and tenacity, unable to stop and reappraise the current triggering event for its true value.

This section will summarize variants of Linehan's theory in particular, because it has provided the rationale for hypotheses in brain imaging studies. Then, it will discuss interpretations of findings from those studies.

### 4.1. Biosocial Developmental Theories

Linehan [3] took note of how unresponsive to psychotherapeutic intervention the* phenomenon* of emotion dysregulation in BPD patients is and she attributed it to a “fundamental” psychological* mechanism* of emotion dysregulation. She conceived the creation of that mechanism in “biosocial” terms, that is, as the outcome of a “biological irregularity … combined with certain dysfunctional environments,” in the manner of neglect or exploitation by the child's caretakers [3, p. 42]. The biological vulnerability consists of extraordinary limbic excitability for danger that shows as quick and intense arousal and slow return to emotional baseline. Based on research available at the time, Linehan attributed this biological vulnerability to innate temperament; however, she allowed for intrauterine and postnatal influences. She entertained that the mechanism of that uncontrollable excitability resembles that of partial complex epileptic seizures or other neurologically based limbic dysfunction. On the other hand, she explained the BPD patients' insufficient modulation of limbic “irregularity” as failure to develop modulation skills through experience, not due to a biological shortcoming. Therefore, such skills could be learned belatedly in psychotherapy; they consist of (a) suppressing one's somatic experiences of emotion and the urge to act defensively and (b) turning attention away from the threat and committing to a fear-free priority.

Linehan attributes BPD patients' failure to acquire modulation skills to invalidating environments of their childhood. Invalidating caretakers do not recognize the extraordinary intensity and duration of emotion as an innate irregularity, which they must modulate with extraordinary patience and effort. Instead, they respond to the child's excitability with erratic, inappropriate, and extreme ways, punishing it or trivializing it, as if the child exaggerated it for other reasons. Children, in turn, are left feeling blamed or disbelieved about the genuineness of their emotions. Linehan founded this theory of invalidating environments and insufficient development of emotion modulation on earlier literature about (a) the harmfulness of a family's “expressed emotion” for a member with mental disorder and (b) disregard for certain temperamental expressions of discontent in early infancy because they contradict cultural ideals of femininity. This interpersonal mechanism of emotional invalidation greatly resembles the mechanism of emotional abuse and neglect described later in the context of attachment theory.

Curiously, Linehan does not address the* psychodynamics of emotion dysregulation* in BPD, that is, how patients appraise* reasons to persist with the emotion and defensive action and reasons to regulate it* and, then, how they choose which to enact. Having skills is only part of that determination. On closer look, the course of emotion dysregulation in BPD suggests that patients labor to reason whether to sustain their emotion or regulate it. BPD patients report becoming recurrently aware of the need to contain their fear and defensive action, but also becoming recurrently driven to persist urgently, before they could find sufficient reasons to enact containment [1]. They fail to stop and think because their reasons to will doing so are still insufficient, not despite having intended so with certainty. The section about attachment theory described how children learn the benefit of containing their emotions, which then, under similar conditions, becomes their reason to will containing their emotions later. The last section of this paper demonstrates the clinical importance of understanding the mechanism of BPD patients' emotion dysregulation in psychodynamic terms. There, the author describes an experimentally tested crisis intervention by means of giving the sufferer reasons to stop and think, nonetheless, while still engaged with the object of fear.

Several authors have adopted Linehan's etiological paradigm of developmental failure to compensate for innate limbic hyperexcitability [4–7]. Some [4] coined the name “hyperbolic temperament” for it. A theory by Gratz and colleagues [5] is notable because it began to amend the construct of innate limbic hyperexcitability, without abandoning it altogether, in order to accommodate the multitude of BPD phenomena that contradict it. One such discrepant phenomenon, in articles cited above, is that fear and commitment to defensive actions during episodes of disorder fluctuate, apparently due to current reappraisals of the danger and one's coping options. Gratz and colleagues themselves focus on another example of phenomena that contradict innate limbic irregularity: preadolescent children with BPD traits often take risks without much vigilance for pain and damage.

Furthermore, along with others [7], Gratz and colleagues noticed that adult BPD patients shift between risk-taking and risk-avoidance. Innate tolerance for risk enhances a person's willingness to endure threat as the means to attaining a desired outcome [38]; in BPD patients, it implies ability to regulate limbic excitability for danger in selected contexts. Indeed, between episodes of disorder, BPD patients choose to expose themselves to danger in two qualitatively different ways that could be aptly described as sensation-seeking and risk-taking [1]. In the instance of sensation-seeking, they periodically expose themselves to harm and exploitation for immediate petty satisfactions, recklessly, with genuine indifference for the long term, therefore, with little fear and little need to contain it. On the other hand, they periodically reinvest in lasting relationships for their long-term needs; then, they mind the risk of betrayal and abandonment constantly. In the latter context, the patients' appraisals of risk of betrayal shift abruptly, which, in turn, determines striking shifts in the patients' threshold of limbic excitability for distress and sacrifices that they must endure.

To explain why the supposedly innate limbic excitability for danger prevails only in certain contexts, Gratz et al. [5] postulate a second innate trait, “disinhibition,” which consists of several “dimensions,” like sensation-seeking and risk-taking. Accordingly, BPD symptoms result from a “synergistic influence” of the two traits. The authors properly cite evidence that a measure of tolerance versus aversion for risk is a heritable trait, with obvious advantages for each preference [38]. But they acknowledge that the correlation between* innate* proclivity for risk-taking and BPD in adulthood is far from established. Finally, they offer no theory for the hypothesized mechanism of “synergy” between innate limbic excitability and innate risk-taking.

### 4.2. The Neurobiology of BPD

Structural, resting functional, and task-related functional neuroimaging in BPD found aberrations in activation or volume in a network that consists of particular regions of the PFC (especially the dorsolateral PFC, DLPFC), the ACC, and the amygdala [7, 39–41]. This restates an earlier summary from reviews of findings from brain imaging to set the stage for expansion to subtler data and, next, for discussion of whether the data support the biosocial theories above. A small hippocampus is another consistent finding, but it is not accompanied by functional neuroimaging data and it is a finding for various mental disorders [7, 39, 40]. One review included hyperactivation of the insula and sparse connectivity between the insula and the ACC as a marker of emotion dysregulation in BPD in particular [42]. Other reviews entertained unilaterally low volume in the left OFP and right ACC as typical of BPD, compared with other disorders with much anxiety and depression [40, 41]. This summary uses “aberration” to avoid terms like alteration, dysfunction, disruption, impairment, and so forth, which imply brain damage or cessation of development. The literature is replete with this kind of implicit interpretation of neuroimaging data as damage and maldevelopment. The paragraphs below will gather evidence and reasons for alternative interpretations.

Various findings are discrepant with the initial summary in various ways. For example, some studies showed no structural or functional aberration from imaging of the prefrontal cortex of BPD patients [16, 17]. In another kind of discrepancy, opposite to expectation, the amygdala showed greater activation in response to effortful regulation of emotion, the latter being evident as prefrontal activation. One very common finding raises doubts about the innateness of limbic excitability in BPD and about the interpretation of brain imaging variations as damage: several brain imaging manifestations of emotion dysregulation in BPD are present also during emotion dysregulation in persons with various mental disorders or no mental disorder, for example, in violent offenders, aggressive psychiatric patients, and uncontrollably anxious or depressed persons with unspecified diagnoses [40]. Finally, clinical remission of such uncontrollable anxiety and depression is consistently accompanied by reversal of the functional neuroimaging variations that those patients share with BPD patients at times of dysregulation [41]. That effect follows treatment with either medication or psychotherapy, but it seems to last longer after psychotherapy. It makes a powerful argument against the prevailing interpretation of neuroimaging findings as evidence of damage or stunted development.

Crocker and colleagues give good reasons for alternative interpretations [40]. Neuroplasticity could be a sufficient explanation for most findings, given the role of all these brain parts in consecutive physiological embodiments of learning. A related principle to remember while interpreting these findings is that neurogenesis, new connections among neurons and myelinization, which result in volume changes, ensue only from behavior that comes to successful closure [24]. Very insightfully, Bush and colleagues hypothesized that “success of [behavioral] regulation … might be correlated with cingulate size” [28, p. 215]. It is reasonable to conclude that the physiology of success is what makes treatment with antidepressants or psychotherapy “neurotrophic,” promoting brain growth and maturation, not the office visit or the antidepressant chemical itself [41]. In that light, this paper proposes the following interpretation of neuroimaging data: brain systems which mediate appraisal of possibilities for goals and timely completion of chosen goals vary in activation and size depending on their history of having done so successfully. Small size of those structures reflects repeated failure to learn how to choose* and then complete* the most compelling among everyday goals, like coping with conflict and betrayal of expectations in important relationships. Such is indeed BPD patients' predicament.

## 5. Psychodynamic Theories of Emotion Dysregulation in BPD

In contrast to biological theories, the psychodynamic ones have conceptually the potential to explain all phenomena of emotion regulation in BPD. They aspire to doing so parsimoniously, by postulating a single mechanism, namely, conflict among reasons for or against regulating the emotion, all generated concurrently in the present [1]. For example, psychodynamic theories promise to postulate and prove* reasons and motives* why patients with BPD sometimes regulate inklings of mistrust and fear very easily, as they do in a phase of infatuation with someone. They similarly promise to explain why in other phases of the same relationship patients must exert much effort to regulate such emotions and they occasionally succeed. For psychodynamic theories, forming the intention to regulate an emotion (if biologically able to do so) is separate from the biological ability to enact it. Normal fluctuations or anomalies in the biology of self-control are one among several reasons to contemplate before making the commitment to do so. People who misjudge their biological* ability* to enact their* intention* to self-regulate discover their attention and effort failing their intention. Even persons with biological shortcomings of emotion regulation are able to do so, if only to a point, with much effort and with help from trusted others. Mental disorder is different from repeatedly failing to control decidedly unwanted behavior. It consists of certain behavior becoming more compelling and concurrently more perilous, unwanted.

The main challenge for psychodynamic theories is to discern* reasons and motives* for such* disorder* of emotion regulation. Indeed, BPD patients experience emotion dysregulation as having reasons to persist despite concurrently having reasons to fear persisting. The challenge for psychodynamic theories is in the fact that some of the reasons and ensuing motives are active implicitly, outside the person's awareness. Psychoanalytical and cognitive-psychological theories have different explanations for the generation of implicit influences in psychodynamic conflict. The author proposes a theory to explain emotion dysregulation in BPD that is composed of well-tested cognitive-psychological concepts about the relation between explicit and implicit motives.

### 5.1. The Psychoanalytical Theory

While studying Linehan's theory, Selby and Joiner Jr. determined that it was necessary to augment it with a decidedly psychodynamic dimension [6]. They determined that disorder was about the purposefulness of emotion, the linkage between emotion dysregulation and behavior dysregulation [41]. They thought that, whatever the person's innate temperament might be,* emotion gains strength from the person's reasons to persist with a current goal* that the emotion propels. In emotion dysregulation of BPD, that goal is invariably to ascertain a threat of betrayal. Tying the strength of emotion to the value of a goal implies that emotion becomes uncontrollable if the person's commitment to that goal becomes compelling and urgent. Selby and Joiner Jr. explain that BPD patients ruminate and catastrophize unnecessarily about failing to manage a threat of betrayal. Thus, they kindle their fear and defensive actions repetitively with “pernicious” results in the manner of a “cascade of emotions.” These authors' insight about the psychodynamics of circularity to the end of mastery is very valuable. Still, the limitation of this theory is that it does not address* reasons* why BPD patients catastrophize about a particular instance of betrayal in a particular relationship.

Patients' unstoppable rumination typically is about making sense of catastrophic betrayal in the past. Alternatively, it is about perceived betrayal in the present, however, over a trivial want or in an insignificant relationship. Psychoanalytical theory explains the urgency with which such rumination intrudes with various unconscious motives, that is, motives which patients do not recognize as active in them presently. One unconscious motive that theory attributes to patients is to urgently master old traumatic betrayal, either retrospectively, in their memory, or by asserting themselves over trivial wants in the present. Such interpretations do not explain why mastering old betrayal in memory and fighting over trivial unfairness become urgent in the particular instance, let alone why without the sufferer's awareness. At other times, BPD patients are mostly oblivious to everyday unfairness and can usually end reminiscing of old trauma at will. To remedy that psychoanalytical limitation, the psychodynamic theory proposed in this paper attributes to patients a motive to ascertain a current threat of grave betrayal in a life-defining relationship. Preoccupation with lesser unfairness and with old trauma derives force* from the need to urgently make sense of the threat that matters singularly in the present*. That urgent need lingers latently and influences conscious priorities implicitly, but patients can be helped to remember having made that linkage consciously. They can remember appraising ways to cope with the current threat as futile because of numerous shortcomings; drawing lessons from old trauma engages the sufferers' attention most because it is the one shortcoming that seems in the sufferer's control to remediate.

It is instructive to note how psychoanalytical theory has attempted to explain patients' unawareness of reasons to pursue mastery of old trauma in memory so relentlessly. It has resorted to a biological, instinct-like mechanism called “completion principle” [37]. Accordingly, learning to finish old failed tasks is physiologically branded in memory as of some priority and once triggered later, it automatically displaces the person's consciously preferred priorities. In fact, this explanation was born in the study of PTSD, where uncontrollable reminiscing of a particular traumatic event is a paramount feature. The empirically testable laws of learning theory contradict the notion of such a completion principle. New lessons are physiologically encoded to be remembered only if successful [20, 24]. Unfinished lessons, too, are similarly encoded if the need for their completion is anticipated consciously [21]. Furthermore, once triggered later, the drive to finish an old lesson is instantly subject to reappraisal and modulation, just like any other motive evoked reflexively. The drive to finish learning how to cope with betrayal takes priority only if it is triggered in the context of consciously needing that lesson urgently.

Some psychoanalytical authors explain emotion dysregulation in BPD with yet another unconscious motive. They describe the patient's relentless, though self-damaging, behavior as “repetition compulsion.” They attribute to patients an unconscious motive to replicate being loved for being inadequate, needy, and submissive, as in earlier mistrusted caretaking relationships. That theory says that survivors of childhood abuse make an “introject” of an abusive caretaking relationship as a preferred outcome to be recreated in later relationships. Then, unbeknownst to themselves, they enact the subordinate's part one-sidedly, repeatedly, to induce the other into the dominant role [42, 43].

Findings from social psychology [44, 45] provide an explanation for how regressive motives may be attributed falsely to a person. In other words, a person's actions may appear to serve a latent motive against one's interests (e.g., to be loved for being exploitable), even opposite to one's declared goal otherwise (to test the other's trustworthiness). Curtis [44] critiques psychoanalytic theories that attribute regressive, no longer wanted behavior to “enduring personality dispositions” which prevail* automatically*, that is, by force unrelated to appraisal of current developments. He believes that such theories show “lack of attention to the influence of current situational variables … on maintaining the current self-organization of behavior” [44, p. ix]. In other words, such theories are blind to interpersonal developments that trigger habitual, unwanted behavior faster than they stimulate the person's reasoning to modulate it. Studies in social psychology [46, 47] have demonstrated how a person's honest intent to change old personality traits may be derailed. People committed to learning new social rules still borrow components from their old behavior, which others then misinterpret as a true, though unspoken, intent to deceive and they treat it accordingly. For those who honestly deny such an unspoken intent to deceive, some in the psychoanalytic tradition would still postulate an unconscious motive to regress.

### 5.2. The Proposed Psychodynamic Theory

#### 5.2.1. The Core Deficit and Pathogenesis

Indeed, the outcome of BPD patients' actions during emotion dysregulation is often exploitation by others, as the outcome of the purported unconscious motive to replicate old abuse would be. However, on closer look, the patients' singular motive that drives their self-defeating behavior is the opposite; it is to ascertain threat of betrayal urgently, so as to avert it [48]. Having had an inkling of betrayal, patients become urgently motivated to prove it. With stealth, hiding their true motive, patients stage hypothetical situations that justify making excessive demands and sacrifices, the kind of events that others misinterpret as intended to replicate being loved for their submissiveness and neediness. The proposed theory explains why patients fail to stop and think [49]. Why do they repeat that testing method relentlessly, despite becoming aware that it defeats their true purpose?

That method is born as the only way that children can test the trustworthiness of caretakers who manipulate the child's operations in order to make their own trustworthiness untestable. Caretakers who want to keep their caretaking prerogatives despite failing their function, for whatever reason (exploitation, inadequacy, etc.), give false reasons for failing and then manipulate the child's ability to prove those reasons false. They manipulate the evidence for their reasons and the evidence that alternative caretakers are trustworthy or the child is worthy of them. If their deception is discovered, they shift the criteria of proof of their fidelity and earn the child's patience with bribes and threats of punishment.

Putting the burden of proving their deception on the child perverts the rules of intimacy [1]. To explain the gravity of that, it is necessary to briefly define the function of intimacy and its rules. Partners in intimate relationships (e.g., parents and their children, siblings, lovers, and life mates) make a commitment to collaborate for the satisfaction of each other's needs in the long term, despite any foreseeable adversity. They commit to taking care of each other's needs before they can know them, even after one partner may become unable to contribute. By the rules of intimacy, a partner who fails promises made and expectations fostered has the burden of proving reasons to fail, including temptations and selfishness. The aggrieved partner, on the other hand, has the burden to make up for the failing partner's legitimate shortcomings and to help correct them, without exploitative tradeoffs and punishments. The rules of intimacy serve to invite partners to reveal their weaknesses and get help, without danger of exploitation or abandonment. Loving partners strive to prove themselves trustworthy, instead of requiring the other partner to catch them dishonest.

Staging hypothetical situations suddenly is the only power that a child has to test caretakers before they could create false appearances of good reasons to fail. At the same time, for children trapped in untrustworthy relationships, that becomes the limit of their imagination about how trust can be measured and tested. Upon having an inkling of betrayal, BPD patients presume that others will necessarily manipulate them. The author proposes that this belief is the core deficit in BPD.

#### 5.2.2. The Testing Method

*Regressive Social Learning [48, 49]*. Having had an inkling of betrayal, BPD patients urgently assume the burden to prove it. With stealth, they stage hypothetical situations that justify making excessive demands and sacrifices. Continually shifting moods of suspicion, vengeance, cajoling, self-blame, and expiation all occur in the context of such testing. According to the author's clinical experience and research cited below, vehement reminiscing and testing lesser relationships occur only in the context of a crisis of trust in a single relationship that matters. Others usually mistake the patient's purpose as manipulation for trivial or inappropriate wants. Some partners take advantage with bad tradeoffs and punishments. The better ones become wary and set harsh limits. For the patient, these painful responses become new triggers of apprehension and any justifications offered by the partner become new obscure reasons to sort out as true or false. The patients' own testing activity renders them less certain than before, one way or the other.

#### 5.2.3. The Theory of Disorder in BPD

The author's theory of mental disorder hinges on a cybernetic concept. In cybernetic terms, disorder consists of derangement of a system's self-correcting mechanism, or “governor” [20, 28]. In human behavior, the governor is stopping to think. It consists of taking a step back to correct errors in the pursuit of a goal or ending the troubled goal unfinished to replace it with a more opportune one. Mental disorder, in turn, consists of errors in the governor's activity itself, which then results in failure to correct the troubled goal as well as failure to replace it. Rethinking the importance and urgency of ascertaining catastrophic betrayal could become a laborious goal of its own priority. The will to stop the troubled goal and think is constructed gradually with reasoning about intervening pains, costs, and competing needs gone unattended. BPD patients' experience is indeed of insufficient reasons, insufficient determination to stop their self-defeating behavior, whereas each round of failure generates new urgency to try again, before the sufferer could stop and think.

## 6. Implications for Treatment of Emotion Dysregulation in BPD

The author has developed a psychotherapy model for BPD and complex posttraumatic disorders. It is the Role Reconstruction Therapy (RRT) model, formerly presented as the Cape Cod Model [50]. It is based on the theory that the patients' regressive testing, commonly recognized as repetition compulsion, is the core deficit or core source of uncontrollable errors in episodes of disorder. The treatment is designed to engage the patient in learning the rules of intimacy. Patients and a partner in a particular troubled relationship, for example, a parent, brother, or lover, stage a few daily commitments to fulfill goals where one or the other anticipates betrayal. Betrayal, in turn, is defined as breaking promises made for bad reasons, namely, (a) for self-serving priorities or (b) because of shortcomings, like deficient skill, forgetfulness, anxiety, and so forth, which the failing person could remediate with others' help. The object of therapy is to demonstrate that it is possible to test another's trustworthiness with some certainty by the rules of intimacy. That entails requiring the failing partner to prove good reasons for the failure, instead of requiring the aggrieved partner to prove hidden bad reasons. This section will describe the crisis intervention according to the RRT and will present results from a study of its efficacy [49].

According to the RRT crisis intervention [49], the therapist first discerns the trigger of hyperarousal, that is, the threat of betrayal in a current relationship of singular importance. Then, the therapist engages the patient in ascertaining that partner's trustworthiness in an effective and timely manner, to replace repetition compulsion. The expected outcome is that the disorder will subside promptly upon engagement in coaching about the rules of intimacy and that the remission will last as long as the patient remains engaged. Patients will not relapse to disorder even if they discover betrayal in the particular instance, because their disorder is about their goal to attain certainty, one way or another.

The crisis intervention provides a technique to sidestep all symptoms (repetitive reliving of old trauma and disputes over trivial conflict in the present) in order to engage the patient about the underlying crisis of trust and repetition compulsion in the single relationship that matters urgently. The clinicians demonstrate how intimacy can be made safe: partners become transparent about reasons to fear and to mistrust, as well as reasons to fail the other's trust, if both parties respond in good will, without recrimination or rejection. This is a method that patients cannot envision on their own, to replace repetition compulsion, the mechanism of disorder and the source of all symptoms. With each crisis intervention, patients internalize a measure of competence in ascertaining threats of betrayal on their own. Beyond containment of symptoms, crises are treated as opportunities for reparative therapy in their own right.

The author tested the efficacy of this crisis intervention [49]. The study measured reduction of symptoms in the experimental and control groups treated separately, at comparable crisis intervention centers (run by the same authority, treating similar populations, and using the same admission criteria). The control group received treatment as usual (TAU, which included elements of DBT and much more medication).

Ordinarily, the experimental intervention takes place for an hour or two initially and then in several shorter sessions over a period of the next day or two. Typically, patients arrive loudly preoccupied with desire, mistrust, worthlessness, and powerlessness in various relationships, including trivial or hallucinated ones. The therapist stimulates that preoccupation by listening empathically and inquisitively, in hope of eliciting associations with the relationship that matters the most, for example, with one's mother, brother, son, or lover. The therapist discerns that object of the patient's rising need and fear and speaks to those emotions with empathy and a hint of hope, for example, saying “it must be insufferable to live with such fear of someone that you need so much. There is a way to become more certain whether your fear is justified.” Invariably, patients respond with a sudden lull in their unstoppable, irrational activity. In that lull, the therapist proposes that there is indeed a better method to become sure of the mistrusted partner's intentions, one way or the other; it can work for one failed commitment at a time, but certainty about the other's overall trustworthiness can accumulate in measurable increments. Engagement in that proposition replaces the patient's frantic regressive testing and symptoms cease for the duration of that engagement.

Modulation of particular symptoms with medication, grounding, and so forth is useful to facilitate engagement and reengagement in the therapeutic proposition. But such measures become unnecessary for hours or days at a time, as long as the patient invites the partner to ascertain each other's trustworthiness by the rules of intimacy: the patient and the partner assume the burden to reveal their true shortcomings for failing promises made and expectations fostered; then, they commit to a reasonable plan to remove those shortcomings jointly, with the aggrieved party's help.

The efficacy study found significant reduction of symptoms within 8–24 hours after initiation of this intervention, primarily as measured with the* Brief Psychiatric Rating Scale (BPRS)*. The BPRS consists of eighteen items and five subscales. There was significant improvement in the total BPRS score for the experimental group. Among the five subscales (thought disorder, withdrawal/retardation, anxiety/depression, hostility/suspiciousness, and activation), improvement was most significant for the four mood-related ones.

The Cochrane Collaboration reviewed studies of efficacy of crisis interventions for patients with BPD and recognized this study as one among 15 studies that “merited closer inspection,” out of 1958 studies screened [51]. Compared to this rapid recovery of thoughtfulness and ability to regulate emotions, other selected “crisis interventions” lasted much longer, up to one month.

## 7. Conclusion

This paper reviewed the literature pertaining to the pathogenesis of emotion dysregulation in BPD and then it proposed a psychodynamic theory that the author argues remedies certain limitations of earlier theories. The author also presented the principles for a psychotherapy model according to the proposed theory.

The strength of this theory, compared to biological ones, is that it explains why limbic hyperexcitability in BPD is only episodic and strictly object-specific; it pertains only to danger of grave betrayal. In that sense it validates the collection of criteria in the DSM as a coherent entity. Compared to psychoanalytical explanations, in turn, the proposed theory has the advantage that it postulates implicit mechanisms and forces which can be verified as psychological facts. There is preliminary evidence that those conceptual advantages are of clinical value, measured as efficacy of treatment. The main limitation of the proposed theory is its status as largely untested. The efficacy of crisis intervention itself could be tested more rigorously, by isolating the effects of the singular RRT intervention (engagement in ascertainment of threat by the rules of intimacy) from other incidental treatment variables more methodically. There has been no investigation of efficacy for the long-term treatment with the RRT.
